# Carcinoma-derived exosomes modify microenvironment

**DOI:** 10.18632/oncotarget.3182

**Published:** 2015-01-22

**Authors:** Norman J. Maitland

**Affiliations:** YCR Cancer Research Unit, Department of Biology, University of York, York, United Kingdom

Secreted vesicles have had a rather a mixed press in cancer biology. Originally considered as a ‘cellular trashcan’, with particular usefulness as a repository for specific proteins from the originating cell type, the literature now abounds with papers describing the usefulness of exosomes: one class of secreted vesicles. In this issue, Chowdhury et al [1] have assigned a potent function to these small (30-100nm) vesicular particles, which carry a variety of macromolecular cargoes, including proteins and various forms of RNA. When secreted from cancer cells into prostate cancer patients’ serum (reviewed in [2]), it is likely that the ‘exosomal signature’ can be exploited as a useful diagnostic tool. Indeed, there are strong arguments that many traditional ‘serum’ and urinary markers for cancer are actually present in the exosomal fraction, which performs not as a trashcan, but more as a protective envelope for labile molecules such as mRNA and miRNA. Good experimental evidence already supports a functional role for RNA transferred by exosomes, but now Chowdhury *et al* show that a more direct effect is transmitted via a common and potent cytokine - Transforming Growth Factor Beta (TGFβ).

The demonstration that exosomes produced by the DU145 prostate cancer cell line (derived originally from a rare brain metastasis) directly influence the phenotype of mesenchymal stem cells (MSCs) *in vitro*, differentiating the MSCs to a more myofibroblastic cell, identifies an additional conversation in the cross-talk which defines the tumor microenvironment. It is known that non-tumor myofibroblastic cells - carcinoma associated fibroblasts (CAFs), can have profound effects on multiple epithelial cell types [3]. However the origin of CAFs has not been defined, certainly in prostate cancers. Chowdhury et al showed that exosomally tethered TGFβ was able to differentiate MSCs into a myofibroblastic cell which promoted carcinoma cell growth and invasion, and increased angiogenic potential. Previously, soluble TGFβ had been shown to induce a cancer-like phenotype, including epithelial to mesenchymal transition (EMT), in the BPH1 cell line (SV40 T antigen-immortalised benign prostatic epithelial cells) [4]. There was however no evidence in Chowdhury *et al*, to implicate an EMT mechanism for the effects of the differentiated MSCs, although the DU145 cells employed in the experiments already possess an aggressive stromal-like (vimentin positive) phenotype. More importantly, whereas soluble TGFβ could influence the MSC phenotype, the effect was considerably greater and more extensive when TGFβ was delivered by exosomes.

**Figure 1:**
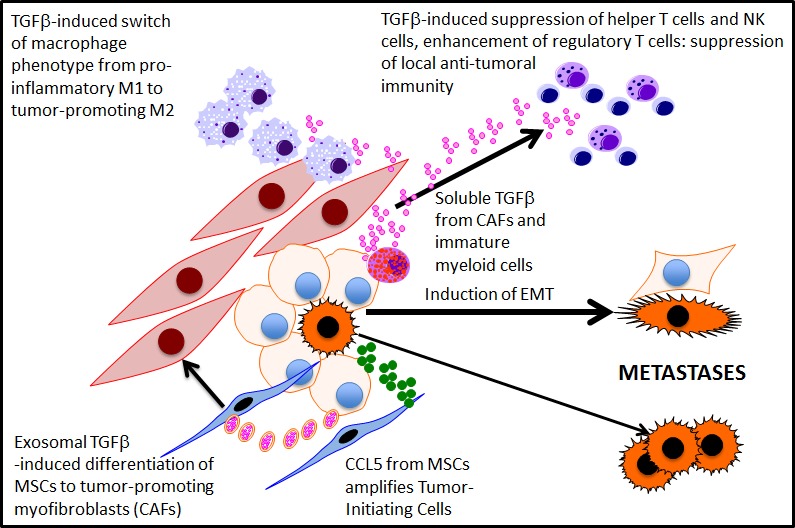
Exosomal and soluble TGFbeta mediated signalling in the tumour microenvironment

TGFβ is a truly pleiotrophic growth factor within the tumor microenvironment whose effects on tumor stromal cells, including fibroblasts and lymphoblastoid cells, have been intensively studied. Not only does it influence stromal phenotype, but the TGFβ originating from a tumor mass also has a potent effect on the local anti-tumour cell immune response [5], and patients with high levels of serum TGFβ (in exosomes?) do substantially worse than those with lower levels [6]. The intercellular communication is therefore multidirectional - as shown in a somewhat simplified form in Figure 1.

Prostate cancers are phenotypically heterogeneous, and like many tumor types contain a minor population of cells with an enhanced tumor initiating capacity (TICs) – also known as cancer stem cells. Recently, Luo et al, [7] demonstrated that the MSCs which infiltrate experimental prostate cancers secrete CCL5 (RANTES), amplifying a TIC population by 2-3 fold. It was assumed that the increased number of TIC cells resulted in enhanced metastasis. Could this effect also be mediated by exosomes? By using an established cell line, cellular heterogeneity may have been eliminated in Chowdhury *et al*. Equally, if the exosomal TGFβ is designed to suppress anti-tumor immunity and to prepare the metastatic site for colonization, then it is perhaps logical to propose that the TICs would secrete the highest number of TGFβ+ exosomes. The reported high serum levels of TGFβ in aggressive prostate cancers (and patients’ serum) [6] relative to the paucity of TICs implies that the effector exosomes are more likely to be a sustaining function of the bulk tumor cells, which allows the tumor to develop by influencing not only the microenvironment, but also other epithelial tumor cells.

Although the same group had previously shown that similar TGFβ+ exosomes are secreted by some but not all common prostate cancer cell lines, it will now be important to extend what is essentially a cell line-based, in vitro study into more exhaustive in vivo studies: does TGFβ do just some or all of the things proposed to nurture tumor growth and development in an animal (or patient) model and - are all tumor cells equal in their exosome production capacity? Finally, there is probably little happening in the tumor microenvironment, which is not related to normal cell or embryonal functions. What is the presumed ‘normal’ role of exosomal TGFβ? In terms of therapy, it is perhaps naïve to think that blocking the phenotypic changes induced by exosomal TGFb, without off-target events, which would affect the normal tissue homeostasis controlled by TGFb growth suppression. As Chowdhury *et al* also reported, the carcinoma-specific downstream effects should present a more cancer-specific target.
